# Comparison of Laminoplasty vs. Laminectomy for Cervical Spondylotic Myelopathy: A Systematic Review and Meta-Analysis

**DOI:** 10.3389/fsurg.2021.790593

**Published:** 2022-01-17

**Authors:** Huaguo Zhao, Rong Ren, Weihu Ma, Song Xu, Linrui Peng, Zhaoping Zhong, Yan Zheng

**Affiliations:** ^1^Department of Orthopedics, Ningbo No.6 Hospital, Ningbo, China; ^2^Department of Hepatobiliary Surgery, Shangyu People's Hospital of Shaoxing, Shaoxing, China

**Keywords:** cervical myelopathy, laminoplasty, laminectomy, meta-analysis, systematic review

## Abstract

**Objectives:**

Laminoplasty (LP) and laminectomy (LC) with or without fusion are recommended as treatment procedures for cervical spondylotic myelopathy (CSM). The purpose of this study is to conduct a meta-analysis to analyze the results of CSM patients undergoing LP or LC surgery.

**Methods:**

We systematically and comprehensively searched Web of Science, Cochrane Library, PubMed, EMBASE, OVID, VIP database, Google Scholar, Chinese Bio-medicine Literature database, and China Scientific Journal Full-text database to July 2021 for randomized controlled trials (RCTs) and observational case series that compared LP and LC in patients with CSM. The main endpoints were the surgical process, radiographic outcomes, clinical outcomes, and surgical complications.

**Results:**

A total of 19 were included the inclusion criteria in this meta-analysis (*n* = 4,348 patients). There was no significant difference in range of motion (ROM), sagittal vertical axis (SVA), Japanese Orthopedic Association (JOA), Cobb angle, visual analog scale (VAS), cervical curvature index (CCI), Nurick score, Neck Dysfunction Index (NDI), and complications. LP was found to be superior than LC in terms of complications of C5 radiculopathy and surperficial infection.

**Conclusion:**

Our results indicate that LP can achieve better results in C5 radiculopathy and superficial infection in surgical treatment of CSM compared with LC. Further high-quality research is warranted to further verify our findings.

**Systematic Review Registration:**

PRISMA: CRD42018107070.

## Background

Cervical spondylotic myelopathy (CSM) refers to a clinical chronic disorder that is usually releated with degenerative disease of intervertebral disk (1). Cervical spondylotic myelopathy is the most common spinal cord degeneration in older sufferers, caused by the progressive spinal canal stenosis and subsequent nerve root compression (2). Surgical management is generally indicated for patients with CSM when conservative treatments are ineffective. Anterior cervical decompression and fusion for multilevel CSM is a complex procedure and may be associated with a long operative time, as well as complications, such as dysphagia, internal graft dislocation, and trigeminal nerve palsy (2). Laminoplasty (LP) and laminectomy (LC) with or without fusion are the primary posterior cervical surgical strategies for treating CSM to remove compressive elements, providing enough space for the cord, and decompressing the spinal cord (3).

Laminectomy, which is usually supplemented by additional fusion, was initially viewed as the gold standard practice for CSM (4). However, this technique is associated with many disadvantages, such as post-LC kyphosis, segmental instability, and subsequent neurological deterioration, which lead to a shorted indication. Laminoplasty was first reported by Tsuji et al. (5) in 1982, and is regarded as an effective way of maintaining anatomical cervical reduction. Laminoplasty retains a covering of the ligamentum flavum over the spinal cord and posterior laminar bone. Laminoplasty has the advantages of minimizing instability, limiting constriction of the dura from extradural scar formation, preserving motion, and avoiding complications related to fusion. However, LP is contraindicated in patients with CSM and >13° of kyphosis and severe neck pain (6). Although there are several disadvantages of LP, including vertebral canal reclosed problems, hinge fracture, higher technical requirements, and possible injuries to the cervical cord, LP has been gradually accepted by an increasing number of surgeons. At present, both surgical procedures decompress the spinal cord by enlarging the spinal canal and are regularly thought to be effective in treating CSM.

Although several meta-analyses or systematic reviews have been conducted to compare LC with LP in treating CSM, the results of these studies were not consistent. In a systematic review, Chen et al. (7) reported that LC with fusion had a higher rate of reoperation, non-union, and infection compared with LP. However, Fehlings et al. (8) suggested that LP and LC with fusion had similar effectiveness. Laminoplasty and LC with fusion may result in clinical recovery and a similar loss of lordosis. Similar to LC followed by fusion, expansive LP has a shorter operative time and less C5 palsy. In this study, we aimed to provide some references for clinical surgical treatment of CSM by systematically comparing the safety and efficacy of LP and LC regarding surgical outcomes, radiographic outcomes, clinical outcomes, and surgical complications.

## Methods

### Literature Search

International prospective register of systematic reviews (CRD42018107070) was prospectively registered in the Preferred Reporting Items for Systematic Reviews and Meta-Analyses (PRISMA) (9) for this meta-analysis. Potentially relevant papers that were published in the Web of Science, Cochrane Library, PubMed, EMBASE, OVID, VIP database, Google Scholar, Chinese Bio-medicine Literature database, and China Scientific Journal Full-text database to July 2021 were retrieved and read. Additionally, we also screened the list of references included in publications and related reviews. All included literature only considers English or Chinese language. The following MeSH terms associated with text words were used: cervical vertebrae, spinal cord compression, LP, and LC.

### Selection Criteria

Two researchers independently judged the eligibility of all studies retrieved from the database. Any disagreements between two independent researchers are resolved through discussion or consultation with a third researcher. This meta-analysis is included in the study based on the following criteria: (1) randomized controlled trial (RCTs) or observational studies; (2) studies that compared clinical outcomes between LP and LC with or without fusion; (3) studies that included patients with CSM caused by spinal stenosis; and (4) studies that included outcome measurements, such as surgical outcomes, radiographic outcomes, clinical outcomes, and surgical complications. The exclusion criteria are as follows: (1) The randomized study did not conduct a control group study; (2) The data in the paper or research report was incomplete, resulting in unclear research results; (3) Incomplete papers, including abstracts, conference reports, case reports, and comments And expert opinions; (4) There is no data in the research results to estimate the relative risk (RR) and mean difference (MD).

According to the Cochrane risk-of-bias criteria, the risk of bias and methodological quality of the involved included studies was evaluated by two researchers independently. The methodological quality of each studies was assessed as unclear risk, low risk, or high risk.

### Data Extraction

All revelant data were extracted independently by two researchers from article texts, tables, and figures. The extracted data includes: the first author, the time of publication, the origin country of the study, the type of experimental design, the sample size of the study, demographics, methods, length of post-operative follow-up and clinical results. The clinical results included length of operation, loss of blood, length of hospital stay, range of motion (ROM), sagittal vertical axis (SVA), Cobb angle, Japanese Orthopedic Association (JOA), mJOA, visual analog scale (VAS), cervical curvature index (CCI), SF-36 MCS, SF-36 PCS, Nurick score, NDI (neck disability index), and C5 radiculopathy.

### Statistical Analysis

Relative risk with a 95% CI was computed for binary data, and the MD with a 95% CI was computed for continuous data. The heterogeneity between studies was assessed by the χ^2^ test and the *I*^2^ statistic. *I*^2^ ≤ 50% indicates acceptable heterogeneity, while *xI*^2^ > 50% means significant heterogeneity. A fixed effects model was applied, if *P* > 0.10 and *I*^2^ <50%. If not, a random effects model was chosen. Publication bias is evaluated by the symmetrical construction of the funnel chart. Review Manager (RevMan 5.3, Cochrane Collaboration, Nordic Cochrane Center, Copenhagen, Denmark) was performed for statistical analysis.

## Results

### Search Results

**Figure 1:**
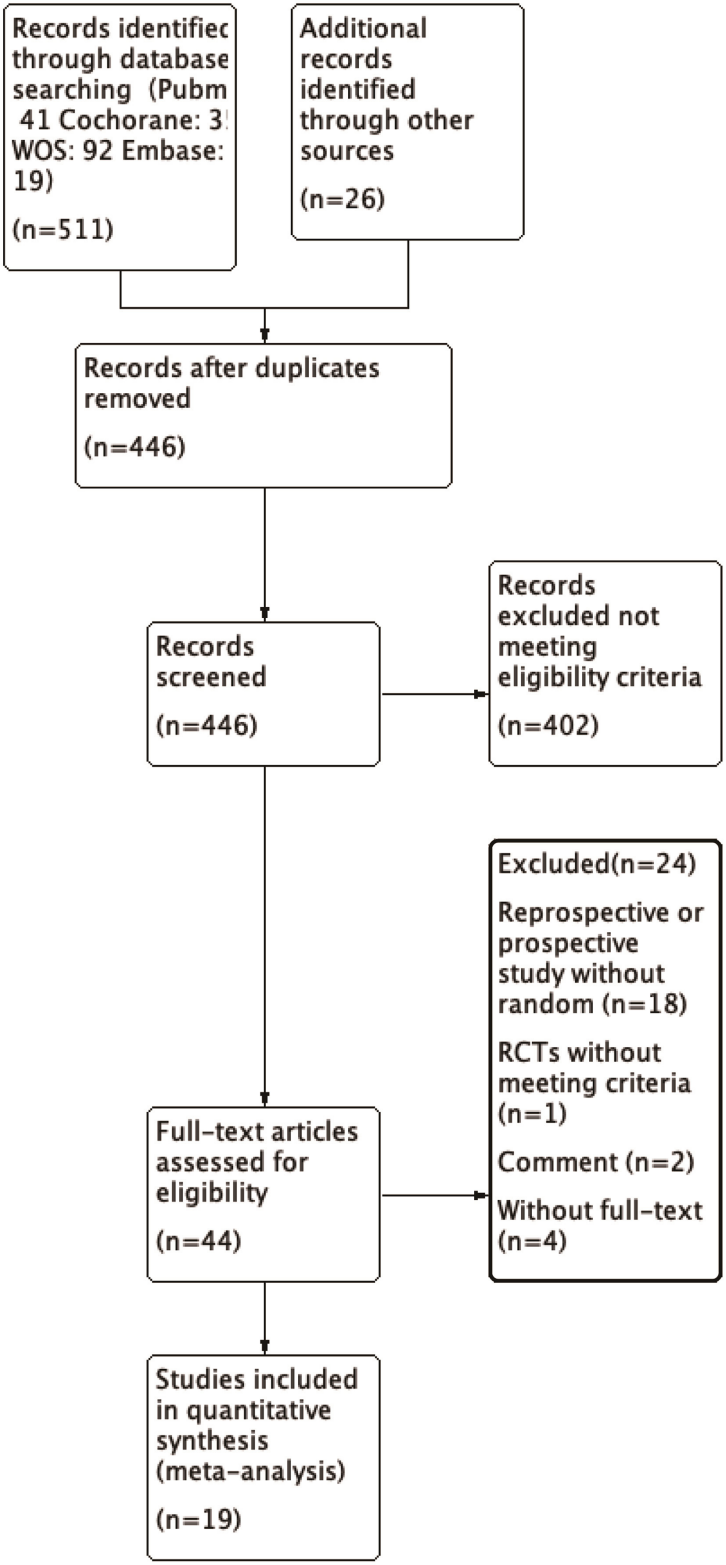
Flowchart of study search and inclusion criteria.

**Figure 2:**
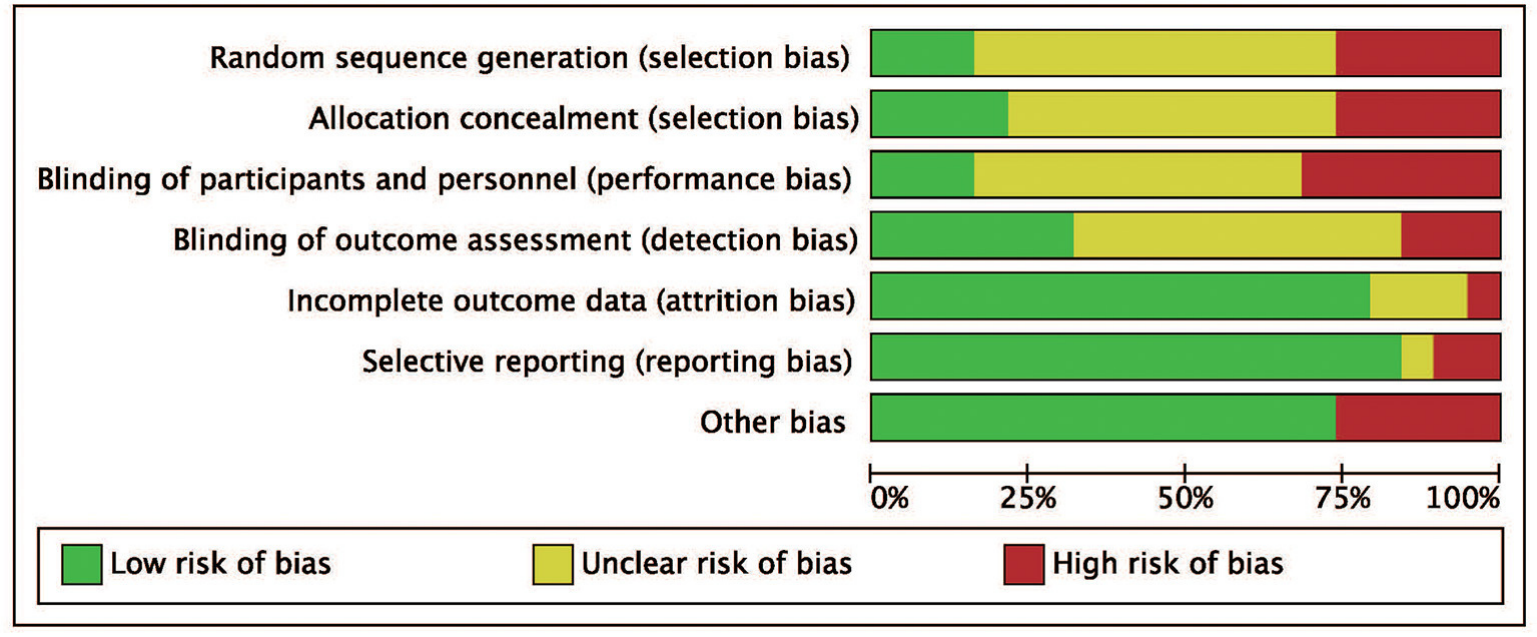
Risk of bias graph. Review authors' judgments for each risk of bias item are presented as percentages across all included studies.

**Figure 3:**
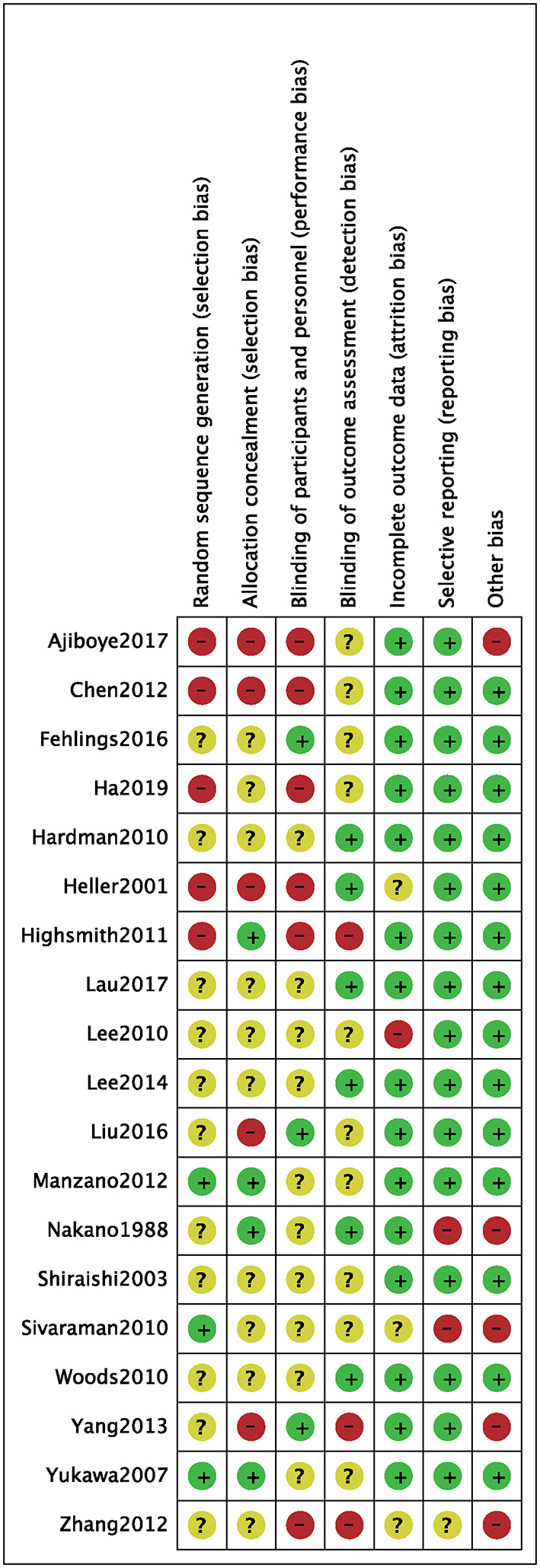
Risk of bias of included randomized controlled trials. +, no bias; –, bias; ?, bias unknown.

We identified a total of 511 probably related articles from the database. An additional 26 possible publications were identified through other sources, primarily through manual search of the reference list. Of the 537 articles, 443 articles were excluded due to duplication or the exclusion criteria. Finally, a total of 19 articles (4, 6–8, 10–24), which were published between 1988 and 2019, were considered for inclusion in this meta-analysis. The detailed search strategy based on database is provided in Figure 1. A total of 1,724 patients (896 treated with LP and 828 treated with LC), were included, and the follow-up period ranged between 8.8 and 72 months. As shown in Figures 2, 3, the results of methodological quality are presented in the risk of bias. Only three studies (12, 17, 25) were RCTs that compared LP with LC. The Adetailed overview of study characteristics can be consulted in Table 1.

**Table 1:** Clinical characteristic of included studies.

**Study**	**Year**	**Study design**	**Simple size**		**Mean age(years)**		**Gender (M/F)**		**Follow-up months**	
			**LP**	**LC**	**LP**	**LC**	**LP**	**LC**	**LP**	**LC**
Ajiboye et al. (23)	2017	PRO	25	45	54.88 ± 9.05	65.36 ± 10.08	19/6	30/15	10.08 ± 12.87	10.67 ± 13.4
Chen et al. (7)	2012	RET	41	32	46.3 ± 2.5	52.6 ± 1.7	33/8	19/13	48–72	48–72
Fehlings et al. (8)	2016	PRO	100	166	60.68 ± 11.32	61.36 ± 10.59	33/67	53/113	24	24
Ha and Shin (24)	2019	RET	49	42	59.12 ± 8.53	62.21 ± 7.81	33/16	36/6	39.61	37.51
Hardman et al. (14)	2010	RET	72	49	59.7	57.3	29/48	14/35	NA	NA
Heller et al. (11)	2001	RET	13	13	56	55	NA	NA	26.2	25.5
Highsmith et al. (4)	2011	RET	30	26	61	58	NA	NA	42.3	41.3
Lau et al. (21)	2017	RET	101	44	63.9 ± 11.9	60.9 ± 9.0	74/27	21/23	17.4 ± 12.3	16.8 ± 8.4
Lee et al. (15)	2010	RET	30	28	52.4	58.6	18/12	19/9	26.2	25.6
Lee et al. (20)	2016	RET	21	15^a^/21^b^	54.2 ± 10.3	• 63.7 ± 6.6^a^ • /63.7 ± 7.7^b^	15/6	• 13/21^a^ • 9/2^b^	8.8 ± 8.4	• 16.8 ± 3.1^a^ • 13.8 ± 11.2^b^
Liu et al. (22)	2016	RET	32	35	59 ± 10	60 ± 8	26/6	25/10	38 ± 13	42 ± 9
Manzano et al. (17)	2012	RCT	9	7	59	61	5/4	2/5	59	61
Nakano et al. (10)	1988	RET	75	14	55.0	59.2	NA	NA	54	128
Shiraishi et al. (13)	2003	RET	51	43	67	69	NA	NA	43	30
Sivaraman et al. (25)	2010	RCT	25	25	62.4	69.6	11/14	13/12	NA	NA
Woods et al. (6)	2010	RET	39	81	60	64	14/25	32/49	23.99 ± 9.91	23.81 ± 5.98
Yang et al. (19)	2013	RET	75	66	57.19 ± 7.33	56.98 ± 8.34	56/19	49/17	NA	NA
Yukawa et al. (12)	2007	RCT	21	20	62.3 ± 11.4	66.1 ± 10.8	13/8	15/5	NA	NA
Zhang et al. (18)	2012	RET	87	56	55.5	58.0	36/51	24/32	NA	NA

*LP, laminoplasty; LC, laminectomy with or without fusion; M, male; F, female; NA, not available; PRO, prospective study; RET, retrospective; RCT, randomized controlled trial; a, laminectomy alone; b, laminectomy with fusion*.

### Clinical Outcomes

#### Length of Operation, Loss of Blood, Length of Hospital Stays

**Figure 4:**
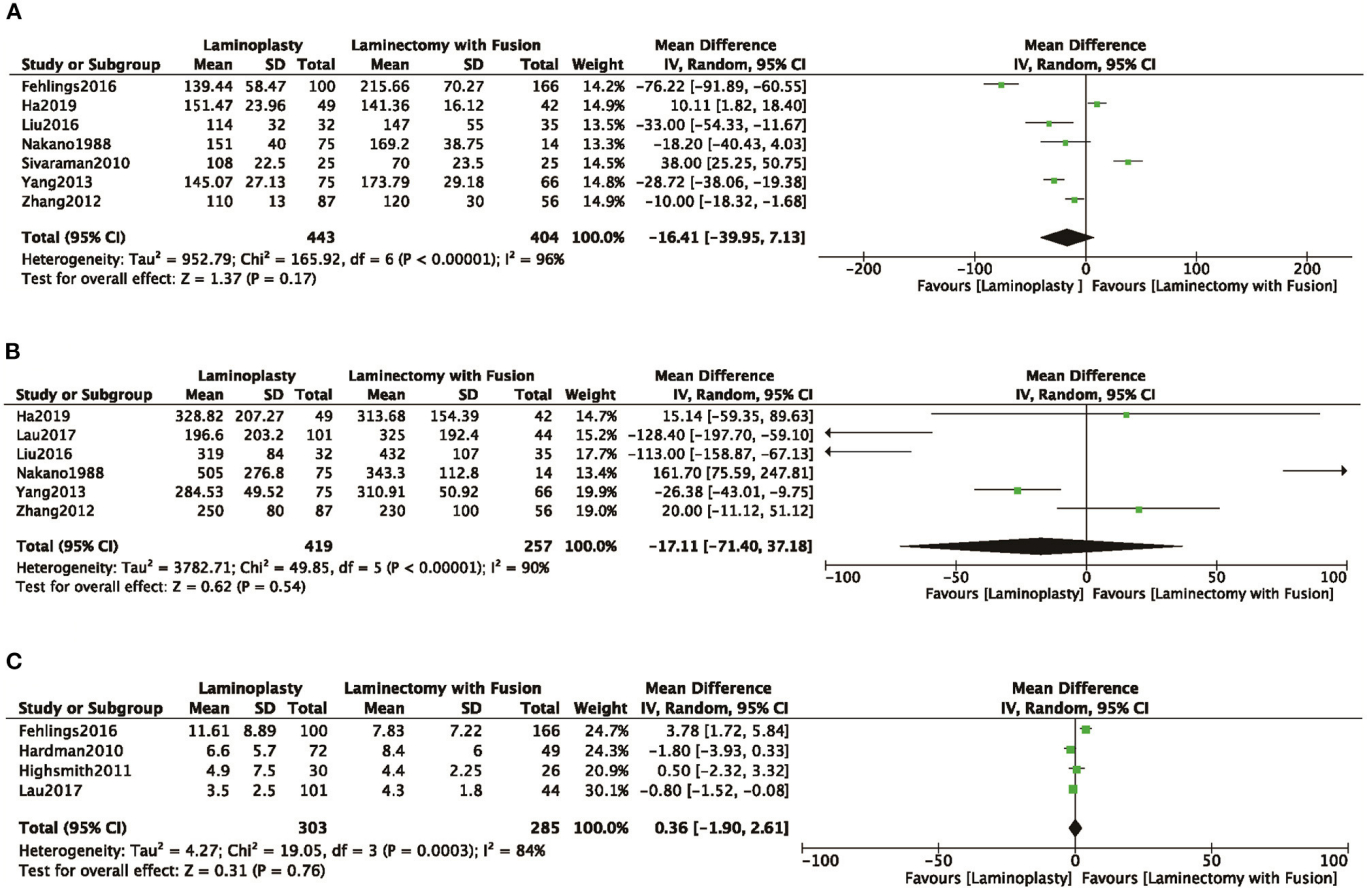
Comparison of **(A)** operative time; **(B)** intraoperative blood loss; **(C)** the time of hospital stay; between the LP group and the LC group.

In seven studies (8, 10, 16, 18, 19, 22, 24) that reported the length of operation, there was no significant difference observed between the length of operation of the LP and LC groups (MD = −16.41, 95% CI: −39.95 to 7.13, *I*^2^ = 96%, *P* = 0.17) (Figure 4A; Table 2). Intraoperative blood loss was evaluated in six studies (10, 19, 21, 22, 24). No significant differences were demonstrated between the volume of blood loss of the two groups (MD = −17.11, 95% CI: −71.40 to 37.18, *I*^2^ = 90%, *P* = 0.54) (Figure 4B; Table 2). The length of hospital stay was evaluated in four studies (4, 8, 14, 21), and there was no significant difference observed between the LP and LC groups (MD = 0.36, 95% CI −1.90 to 2.61, *I*^2^ = 84%, *P* = 0.76) (Figure 4C; Table 2).

**Table 2:** Detail characteristic of included studies.

**Analysis item**	**Studies**	**Patients**	**Heterogeneity**		**Statistical method**	**Effect estimate**	***P*** **-value**
			***I***^**2**^ **(%)**	***P***			
**Clinical outcomes**							
Operative time	7	847	96	0.00	MD (IV, random, 95% CI)	−16.41(−39.95, 7.13)	0.17
Blood loss	6	676	90	0.00	MD (IV, random, 95% CI)	−17.11 (−71.40, 37.18)	0.54
Hospital stay	4	588	84	0.00	MD (IV, random, 95% CI)	0.36 (−1.90, 2.61)	0.76
**Radiographic outcomes**							
ROM	4	416	94	0.00	MD (IV, random, 95% CI)	3.50 (−3.74, 10.73)	0.34
SVA	4	345	75	0.00	MD (IV, random, 95% CI)	−0.78 (−6.34, 4.79)	0.78
Cobb angle	4	345	91	0.00	MD (IV, random, 95% CI)	−1.44 (−6.57, 3.68)	0.58
**Functional outcomes**							
JOA	8	766	71	0.00	MD (IV, random, 95% CI)	0.49 (−0.01, 0.98)	0.06
VAS	8	616	90	0.00	MD (IV, random, 95% CI)	−0.62 (−1.39, −0.15)	0.12
CCI	4	293	86	0.00	MD (IV, random, 95% CI)	−0.17(−2.08, 1.73)	0.86
Nurick score	4	345	91	0.00	MD (IV, random, 95% CI)	−1.44 (−6.57, 3.68)	0.58
NDI	4	605	70	0.00	MD (IV, random, 95% CI)	−1.35 (−3.66, 0.95)	0.25
**Complications**							
Hardware failure	3	348	0	0.71	RR (M-H, fixed, 95% CI)	0.52 (0.13, 2.03)	0.34
C5 radiculopathy	9	931	0	0.52	RR (M-H, fixed, 95% CI)	0.35(0.20, 0.61)	0.00
Adjacent segment degeneration	2	292	0	0.78	RR (M-H, fixed, 95% CI)	0.23 (0.03, 1.95)	0.18
Dural tear	6	701	0	0.51	RR (M-H, fixed, 95% CI)	0.68 (0.27, 1.69)	0.41
Deep infection	2	292	0	1.00	RR (M-H, fixed, 95% CI)	0.33 (0.04, 2.93)	0.32
Superficial infection	7	792	0	0.65	RR (M-H, fixed, 95% CI)	0.45 (0.20, 0.98)	0.04
Dysphagia	2	386	0	0.74	RR (M-H, fixed, 95% CI)	0.45 (0.05, 3.94)	0.47
New radiculopathy (not C5)	2	322	0	0.74	RR (M-H, fixed, 95% CI)	0.44 (0.07, 2.72)	0.38
Postoperative kyphosis	5	720	15	0.32	RR (M-H, fixed, 95% CI)	1.24 (0.51, 3.01)	0.63
Neck/arm pain	5	674	52	0.08	RR (M-H, random, 95% CI)	0.77 (0.48, 1.23)	0.28
Pseudarthrosis	3	291	0	0.34	RR (M-H, fixed, 95% CI)	0.18 (0.03, 1.24)	0.08

*ROM, range of move; SVA, postoperative C2–7 sagittal vertical axis; JOA, Japanese Orthopedic Association; NDI, neck dysfunction index; CCI, cervical curvature index; MD, mean difference; RR, risk ratio; IV, inverse variance; M-H, Mantel-Haenszel; CI, confidence interval*.

#### Radiographic Outcomes

**Figure 5:**
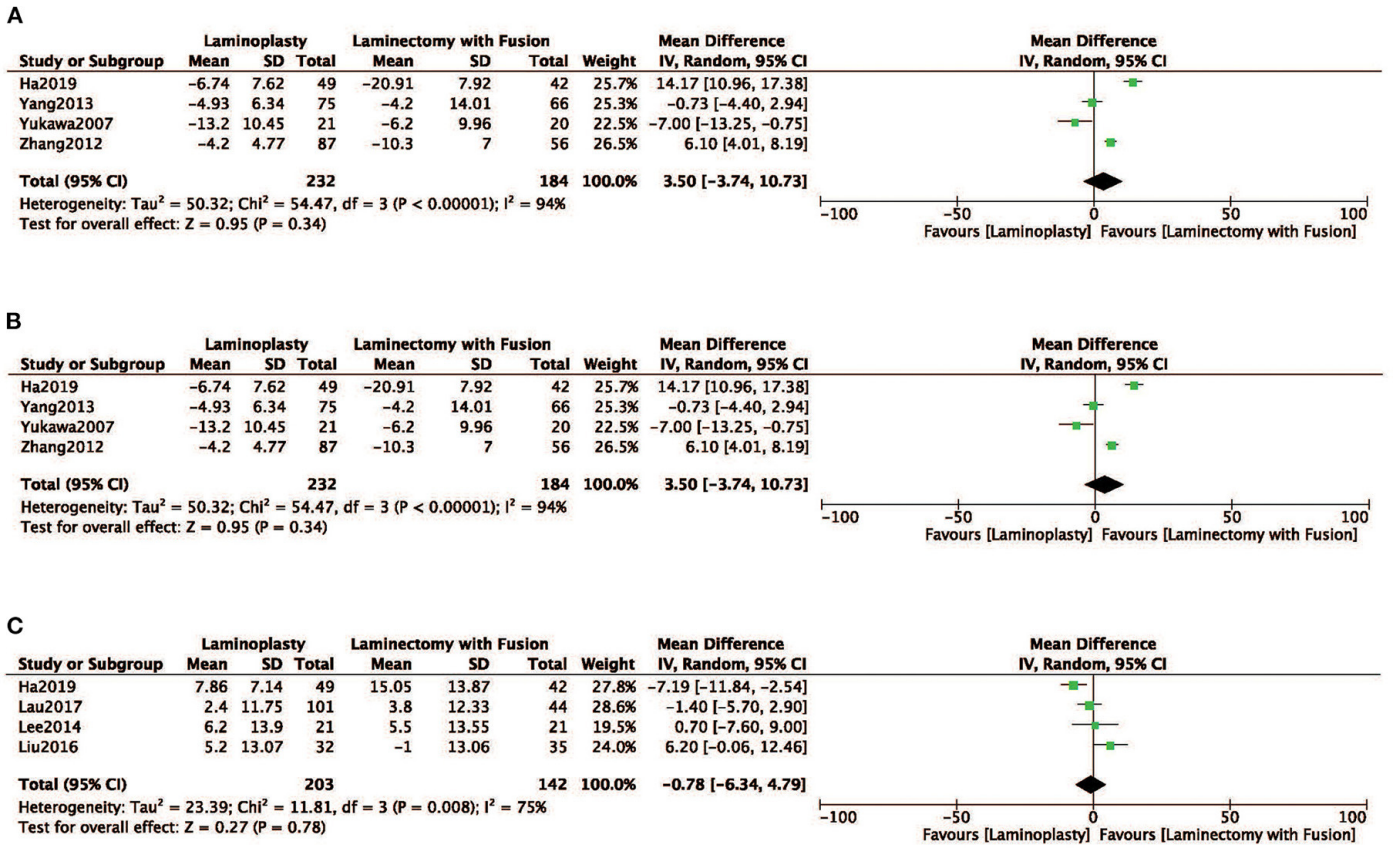
Comparison of **(A)** ROM; **(B)** SVA; **(C)** Cobb angle; between the LP group and the LC group.

Radiographic parameters of the ROM, SVA, and the Cobb angle were evaluated in four studies (12, 18, 19, 24). No obvious difference was observed in ROM between the two groups the LP group compared with the LC (MD = 3.50, 95% CI: −3.74 to10.73, *I*^2^ = 94%, *P* = 0.34) (Figure 5A; Table 2). There was no significant difference observed in SVA between the two groups (MD = −0.78, 95% CI: −6.34 to 4.79, *I*^2^ = 75%, *P* = 0.78) (Figure 5B; Table 2). There was also no significant difference in the Cobb angle observed between the LP and LC (MD = −1.44, 95% CI: −6.57 to 3.68, *I*^2^ = 91%, *P* = 0.58) (Figure 5C; Table 2).

#### Functional Outcomes

**Figure 6:**
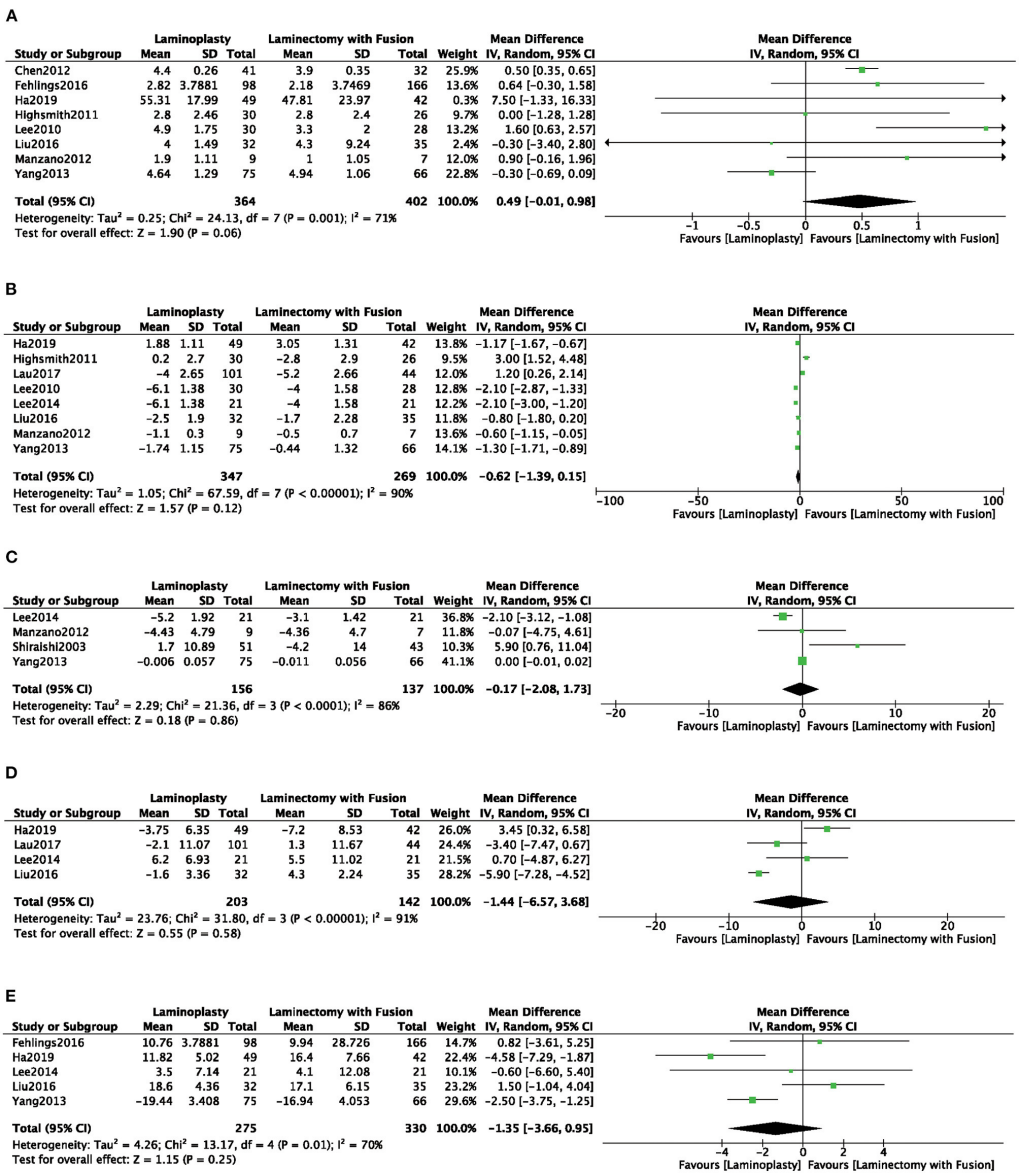
Comparison of **(A)** JOA; **(B)** VAS; **(C)** CCI; **(D)** Nurick; **(E)** NDI; between the LP group and the LC group.

Eight studies (4, 7, 8, 15, 17, 19, 22, 24) used the JOA to assess the clinical outcome. No significant difference was observed of JOA score in the LP group compared with the LC group (MD = 0.49, 95% CI: −0.01 to 0.98, *I*^2^ = 71%, *P* = 0.06) (Figure 6A; Table 2). There were no significant differences in the VAS, CCI, Nurick, and NDI scores between the two groups (Figures 6B–E; Table 2).

#### Surgical Complications

**Figure 7:**
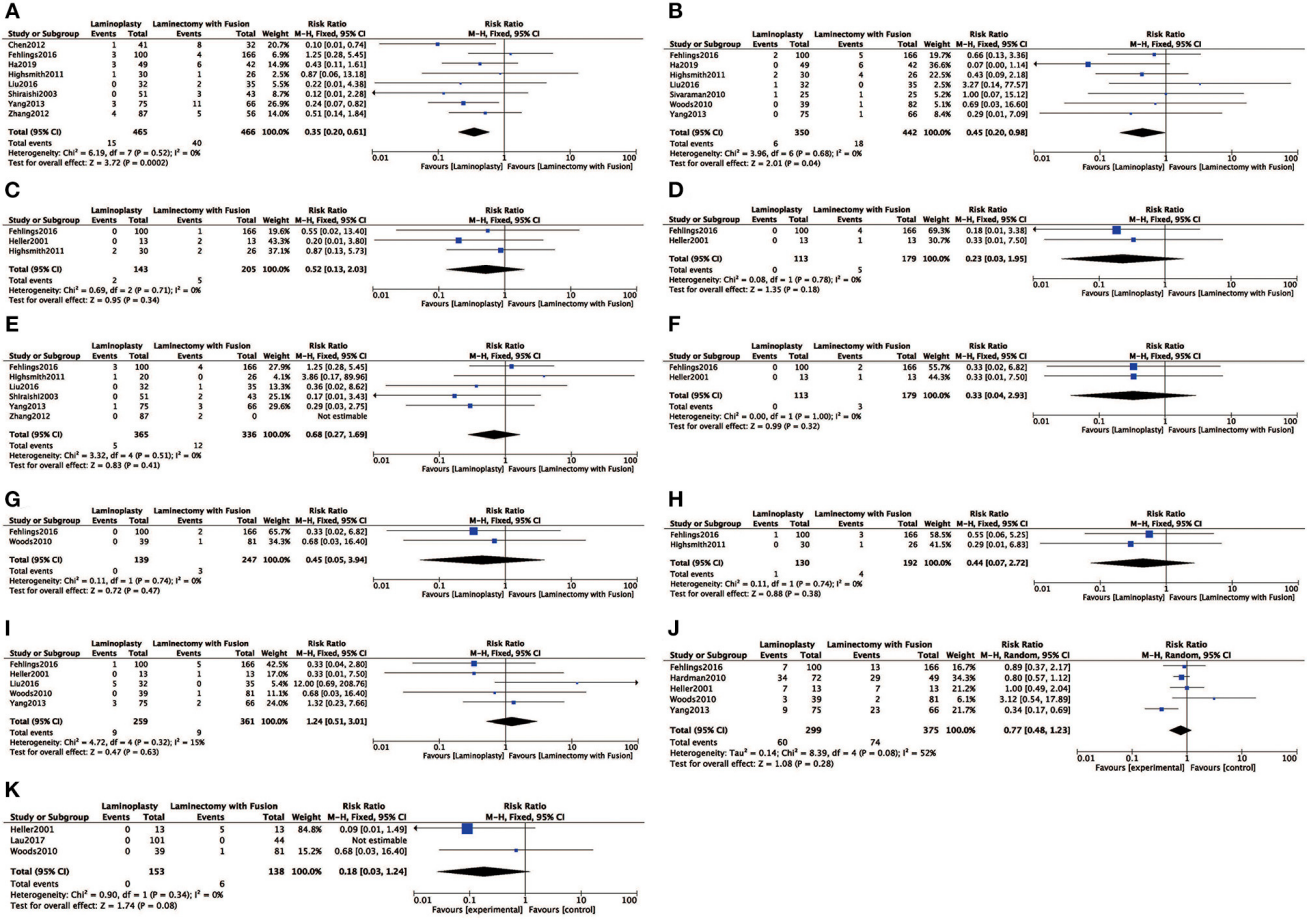
Comparison of **(A)** complication rate of C5 radiculopathy; **(B)** superficial infection; **(C)** hardware failure; **(D)** adjacent segment degeneration; **(E)** dural tear; **(F)** deep infection; **(G)** dysphagia; **(H)** new radiculopathy (not C5); **(I)** postoperative kyphosis; **(J)** neck/arm pain; **(K)** pseudarthrosis; between the LP group and the LC group.

Eight studies (4, 7, 8, 13, 18, 19, 22, 24) evaluated the rate of C5 radiculopathy. A significantly higher complication rate of C5 radiculopathy was observed in the LC gcompared with the LP (RR = 0.35, 95% CI: 0.20 to 0.61, *I*^2^ = 0%, *P* < 0.01) (Figure 7A). C5 radiculopathy occurred in 17 (3.51%) of 485 patients who were treated with LP and in 40 (8.20%) of 488 patients who were treated with LC. Moreover, the complication of superficial infection occurred significantly less in the LP compared with the LP frequently (RR = 0.65, 95% CI: 0.20 to 0.98, *I*^2^ = 0%, *P* = 0.04) (Figure 7B). Additionally, other complications, such as hardware failure, adjacent segment degeneration, dural tear, deep infection, dysphagia, non-C5 radiculopathy, postoperative kyphosis, neck/arm pain, and pseudarthrosis, were not significantly different between the two groups (Figures 7C–K).

## Discussion

Our study showed that patients receiving LP for CSM had less frequent occurrence of C5 radiculopathy and superficial infection than those who underwent LP. Length of operation, loss of blood, length of hospital stays, Cobb angle, SVA, VAS score, CCI score, Nurick score, NDI score, and other surgical complications were not different observed between these two groups. These results suggest that LP is a useful therapeutic procedure promoting management of CSM.

Several previous systematic reviews and meta-analyses that analyzed LP and LC with or without fusion for CSM have been published and showed different results compared with our study (26, 27). A meta-analysis by Lee et al. (28) compared LP with LC for treating CSM. In this article, a total of seven studies were included in the meta-analysis (six English papers and one Chinese paper). The authors focused on the clinical and radiological outcomes between these two different methods. The two groups did not show significant differences in JOA grade, VAS score, and CCI at the baseline state. The authors suggest that both methods may obtain clinical improvement and lead to a similar loss of lordosis, but definitive conclusion could not be reached regarding which surgical approach is more effective for the treatment of CSM. Liu et al. (29) presented a meta-analysis of 23 studies comparing LP with LC for treating CSM. They focused on clinical outcome (JOA, CCI, VAS, and cervical lordosis), complication (C5 palsy and axial pain), blood loss, and operation time. The LP group showed shorter operation time and fewer C5 palsy. Others had may achieve clinical improvement and a similar result. Phan and Scherman et al. (30) compared LP with LC for treating CSM in 10 studies when treating patients with CSM. They focused on the Postoperative JOA, postoperative VAS neck pain, postoperative CCI, postoperative Nurich grade, complication (reoperation rate and nerve palsy), operative time, and intraoperative blood loss. They found that there was no difference in terms of clinical improvement. However, a higher complication of nerve palsy was found in the LC than in the LC. Otherwise, results of others studies showed that LP and LC fusion methods were similarly useful (31).

Patients were matched and both groups had a similar length of operation, blood loss, and hospital stay. These characteristics were not examined in other relevant meta-analyses. Heterogeneity in our meta-analysis was high (*I*^2^ > 75%), those personal difference and surgical processes had multivariate analyses and reports for them are speculative. Pooled results from three studies (21, 22) showed that the Cobb angle was more acceptable in the LP than in the LC. The reason why the Cobb angle was more acceptable in the LP group may be that the muscles and ligamentous structures of the cervical spine are dissected minimally and restored maximally in the LP group. In our study, clinical measurements, such as VAS, CCI, SF-36 MCS, SF-36 PCS, Nurick, and neck disability index scores, were not significantly different in two groups. However, the LP procedure was superior to the LC procedure in evaluation of the JOA score for patients with CSM. These findings indicated that the effect of LP was more favorable than that for LC, which suggested that LP could be considered as the method of surgery for patients with CSM. Most importantly, these results are supportive of a significant improvement in the management of CSM. Specifically, LP as a treatment strategy for CSM can obtain better results in the surgical process, radiographic outcomes, and clinical outcomes (Cobb angle, JOA score, and risk of C5 radiculopathy) than LC.

Several limitations must be considered when interpreting the results. First, the quality of the studies included 3 RCTs and 16 observational studies was low. Most of the RCTs did not provide any insufficient information on the exact methods of randomization to determine if credibility analysis was present. Allocation concealment was performed in three studied using sealed envelopes. Based on the selection criteria, we could not make further deletions or additions to the included papers. Second, the number of individuals in these studies were relatively small and therefore statistical power might be limited. Third, we guess that the research heterogeneity is mainly caused by the low quality of the included articles, which is mainly reflected in the high heterogeneity of the articles. The heterogeneity among the indicators included in our meta-analysis was mainly reflected in the time of operative, intraoperative blood loss, the time of hospital stay, ROM, SVA, Cobb angle, JOA, VAS, CCI, Nurick NDI, and Pseudarthrosis, and for the above heterogeneity, we used a random effects model for treatment statistics, while rate of C5 radiculopathy, Superficial infection, Hardware failure, Adjacent segment degeneration, Dural tea, Deep infection, Dysphagia, New radiculopathy (not C5), Postoperative kyphosis, and Neck/arm pain had lower heterogeneity and we used a fixed effects model for the treatment statistics. In addition, this general clinical heterogeneity may be caused by individual differences in patients, technical differences in the surgeon team, differences in medical equipment, and follow-up time. We considered whether our results might be affected by confounding factors. Therefore, high-quality RCTs are required to examine the long-term effects of these two surgical procedures on patients with CSM.

## Conclusion

Our study shows that LP can achieve better results in C5 radiculopathy and superficial infection in surgical treatment of CSM compared with LC. These results suggest that LP is a therapeutic procedure for promoting management of CSM. Furthermore, high-quality research, adequately powered randomized studies are required to provide more evidence for the optimal surgical treatment of CSM for definite conclusions.

## Data Availability Statement

The original contributions presented in the study are included in the article/supplementary material, further inquiries can be directed to the corresponding author.

## Author Contributions

ZZ and YZ: protocol and project development. LP: data collection or management. WM and SX: data analysis. HZ and RR: manuscript writing and editing. All authors contributed to the article and approved the submitted version.

## Conflict of Interest

The authors declare that the research was conducted in the absence of any commercial or financial relationships that could be construed as a potential conflict of interest.

## Publisher's Note

All claims expressed in this article are solely those of the authors and do not necessarily represent those of their affiliated organizations, or those of the publisher, the editors and the reviewers. Any product that may be evaluated in this article, or claim that may be made by its manufacturer, is not guaranteed or endorsed by the publisher.
